# Microglia exhibit a dynamic response, modulating inducible nitric oxide synthase expression and the production of pro-inflammatory cytokines during experimental cerebral malaria

**DOI:** 10.3389/fimmu.2025.1494418

**Published:** 2025-07-23

**Authors:** Lucas Freire-Antunes, Uyla Ornellas-Garcia, Marcos Rangel-Ferreira, Mônica Lucas Ribeiro-Almeida, Leonardo José Moura Carvalho, Cláudio Tadeu Daniel-Ribeiro, Flávia Lima Ribeiro-Gomes

**Affiliations:** Laboratório de Pesquisa em Malária, Instituto Oswaldo Cruz and Centro de Pesquisa, Diagnóstico e Treinamento em Malária (CPD-Mal) of Fundação Oswaldo Cruz (Fiocruz) and of Secretaria de Vigilância em Saúde (SVS), Ministério da Saúde, Rio de Janeiro, Brazil

**Keywords:** experimental cerebral malaria, macrophages, malaria, microglia, nitric oxide, IL-1β, iNOS, TNF

## Abstract

Microglia play a fundamental role in maintaining central nervous system homeostasis by monitoring brain tissue for physical, structural, and biochemical alterations. Its involvement in the pathogenesis of various neurological disorders is well documented. However, the role of microglia in cerebral malaria, a disease associated with high mortality and long-term neurological sequelae, remains poorly understood. In this study, we utilized the classical model of experimental cerebral malaria (*Plasmodium berghei* ANKA-infected C57BL/6 mice) to investigate the dynamics and response of resident brain cell populations, particularly microglia, and the influx of other leukocytes during the development of experimental cerebral malaria. By employing flow cytometry and established markers for different leukocyte populations, we were able to discern and document an increase in the number of Ly6C^+^ T cells (CD45^hi^CD11b^-^CD3^+^ cells), inflammatory monocytes (CD45^hi^CD11b^+^TMEM119^-^CD206^-^ cells), resident macrophages (CD45^hi^CD11b^+^TMEM119^-^CD206^+^ cells), and microglia (CD45^low^CD11b^+^ TMEM119^+^CD206^-^ cells) following infection. Moreover, our *ex vivo* analysis demonstrated an increment in the overall number of inflammatory monocytes, resident macrophages and microglia expressing inducible nitric oxide synthase (iNOS), in addition to those producing interleukin-1β or TNF. These findings highlight the pronounced reactivity of microglia in experimental cerebral malaria and provide valuable information on cell dynamics and immune responses in the brain.

## 1 Introduction

Despite advancements in the diagnosis and treatment of cerebral malaria (CM), this severe form of the disease continues to represent a significant public health challenge. CM is a complication associated mainly with infection by the protozoan parasite *Plasmodium falciparum* infection, affecting primarily children under five years of age and nonimmune individuals (1). The condition presents as an encephalopathy, which is characterized by seizures and coma (2). Despite treatment, the mortality rate for patients with this complication exceeds 20% (3, 4). However, even if an individual’s CM episode is reversed, they may still experience neurological and cognitive sequelae that can persist for years (5).

The data derived from post-mortem studies in humans (6, 7) and the extrapolations based on studies employing experimental cerebral malaria (ECM) models (8, 9) indicate that CM results from a complex interplay of mechanisms that perpetuate a vicious cycle. The key mechanisms involved include the adhesion of infected red blood cells (iRBCs) to the endothelium of microvessels in the central nervous system (CNS) (6, 8) and the production of systemic and local inflammatory mediators (10). These mediators activate endothelial cells and increase the expression of adhesion molecules, thereby facilitating the recruitment of immune cells to the brain and exacerbating RBC adhesion (11, 12). This can result in microvascular obstruction, hypoxia, and disruption of the blood-brain barrier (BBB) (13, 14).

In addition to the migration of cells from the innate and adaptive immune systems to the brain, which coincides with the onset of neurological signs of ECM and has been documented in post-mortem analyses of CM patients (7, 15–17), the literature also explores the role of resident immune cells of the brain parenchyma, such as microglia, and their involvement in the course of brain disease (18, 19). Microglia are derived from embryonic yolk sac precursors that occupy the embryonic region of the CNS during the early stages of embryogenesis, preceding the formation of the BBB (20, 21). Following the embryonic period, during homeostasis, these cells are able to maintain their constant population through self-renewal, independently of the influx of blood monocytes (22). Microglia play a crucial role in numerous physiological processes, including neurogenesis (23), blood vessel development (24), and the maintenance of the BBB (25). Furthermore, these cells are under constant surveillance, detecting and responding to alterations in the microenvironment, and are essential for the removal of cellular debris and metabolic waste (26, 27).

Morphological alterations in microglia have been documented days prior to the emergence of ECM, indicating that these cells possess the capacity of perceive and promptly respond to changes in the host environment driven by infection (28). The transcriptomic analysis of microglia conducted by Capuccini et al. (29) indicates that in the brains of mice subjected to *P. berghei* ANKA-induced ECM, these cells respond to infection by initially activating cell cycle pathways. As ECM signs become apparent, there is an increase in the expression of genes associated with immune responses and chemokine production. Furthermore, an *in vitro* study has demonstrated that microglial interaction with *P. berghei* ANKA-iRBCs results in the production of TNF and interferon gamma inducible protein 10 (IP10) (18). Additionally, the incubation of synthetic hemozoin with a microglia cell line has been demonstrated to induce the production of TNF, IL-6, IL-1β, and nitric oxide (NO) (30).

The present study, conducted with the *P. berghei* ANKA-C57BL/6 murine ECM, aims to expand the knowledge base regarding the immune cells present and flowing into the brain parenchyma in a temporal kinetic that precedes the development of ECM and during its establishment. Our observations indicated a notable accumulation of T cells and inflammatory monocytes concomitant with the onset of ECM. Additionally, our findings revealed a significant increase in the number of microglia, inflammatory monocytes, and resident macrophages expressing iNOS and producing IL-1β or TNF.

## 2 Materials and methods

### 2.1 Mice, parasite, and infection

Six-to-eight-week-old female C57BL/6 mice, weighing between 16 and 20g, were provided by the Institute of Science and Technology in Biomodels (ICTB) of the Oswaldo Cruz Foundation (Fiocruz). Mice were housed in a specific pathogen-free room at the Oswaldo Cruz Institute (IOC), with free access to food and water, kept on a 12/12-hour light/dark cycle and at a constant temperature. All animal experiments were conducted in accordance with the guidelines and regulations set forth by the Animal Welfare Committee of the IOC-Fiocruz and were approved by the Committee. The experiments were performed under license (L-029/2020).

Infections were performed using *P. berghei* ANKA parasite that express green fluorescent protein (GFP; MR4 number: MRA-865) (31). The experimental group was infected intraperitoneally with 1 × 10^6^ iRBCs in a final volume of 100 µl per animal, with the source of the iRBCs being fresh blood from an infected mouse.

### 2.2 Parasitemia and temperature

On days 4 and 6 after infection, parasitemia and rectal temperature of the mice in the experimental groups were evaluated. The uninfected mice (Control group) were evaluated in parallel. Parasitemia level was determined via flow cytometric analysis of a diluted blood sample, prepared in phosphate-buffered solution (PBS). The percentage of iRBCs (GFP^+^ cells) was calculated after acquiring data from 20,000 RBCs. A thermocouple probe (Oakton^®^ Acorn TM; Oakton Instruments, IL, USA) was utilized for measuring rectal temperature.

### 2.3 Blood-brain barrier permeability assay

To evaluate the BBB permeability, mice were anesthetized intraperitoneally with a combination of ketamine (100mg/kg) and xylazine (10mg/kg), using a final volume of 100 µl per animal. Subsequently, 100 µl of a 2% solution of Evans Blue dye (Sigma) in PBS was injected intravenously through the orbital sinus. After one hour of dye circulation, the mice were euthanized and perfused transcardiacally with 20 mL of cold PBS. The brains were collected, weighed, and incubated in 3 mL of Formamide (Sigma) for 48 hours at 37°C. The concentration of Evans Blue dye in the formamide solution was determined by measuring its absorbance at a wavelength of 620 nm using a spectrophotometer. Calculations were based on a standard curve with a range of 1285 to 1.25μg/mL. The amount of dye was divided by the weight of the animal brain to express the concentration of dye per gram of tissue.

### 2.4 Tissue processing

Following the administration of anesthesia, mice were euthanized by exsanguination followed by transcardiac perfusion with 20 mL of cold PBS. The brain of each animal was removed, weighed and mechanically dissociated using the plunger of a syringe and a 70 µm cell strainer (Falcon) in 20 mL of PBS supplemented with 5% fetal bovine serum (FBS). The cell suspension was then centrifuged for 10 minutes at 500 g, after which the cells were resuspended in 5 mL of PBS containing 30% isotonic Percoll solution (Sigma) at room temperature. A new centrifugation was then carried out for 10 minutes at 700 g without break, at room temperature. The myelin layer was removed with a *Pasteur* pipette, and the cells in the *pellet* were washed three times with 10 mL of PBS containing 5% FBS.

### 2.5 Immunophenotyping

The cells obtained following the processing of brain tissue were incubated with the Live/Dead Fixable Violet Dead Cell Stain Kit (Invitrogen), in accordance with the manufacturer’s instructions. Subsequently, an incubation step was performed with a pool of antibodies, including anti-FCγR III/II (CD16/32) (2,4G2, BD Biosciences), Alexa Fluor 700 anti-mouse CD11b (M1/70, BD Biosciences), FITC anti-mouse CD45 (30F11, BD Biosciences), Texas-Red anti-mouse F4/80 (T45-2342, BD Biosciences), PE anti-mouse CD3 (145-2C11, Invitrogen), APC-Cy7 anti-mouse Ly6C (AL-21, BD Biosciences), PerCP-eFluor 710 anti-mouse TMEM119 (V3RT1GOsz, Invitrogen) and/or PeCy7 anti-mouse CD206 (MR6F3, Invitrogen) for 30 minutes at 4°C in the dark. After immunolabeling of the surface molecules, cells were fixed and permeabilized with the Fixation/Permeabilization Kit (BD Biosciences) and incubated for 40–45 minutes at room temperature with the following fluorochrome-conjugated antibodies for intracellular targets: APC-eFluor 780 anti-mouse NOS2 (CXNFT, Invitrogen), APC anti-mouse IL-1β (NJTEN 3, Invitrogen) and PE anti-mouse TNF (MP6-XT22, BD Biosciences) diluted in perm/wash solution. Samples were acquired on a CytoFLEX S flow cytometer (Beckman Coulter) and the data was analyzed using the FlowJo Software (BD Biosciences).

### 2.6 Statistical analysis

All statistical analyses were performed using Prism 8 (GraphPad). Data are presented as means with standard error of the mean (mean ± SEM). Survival rates were analyzed using the Long-Rank (Mantel-Cox) test. Comparisons between two groups were made by unpaired t-test assuming statistical significance as p<0.05. Comparisons between more than two groups were made by one-way ANOVA with Tukey’s multiple comparisons test. The criterion for determining a significant difference was set at p < 0.05.

## 3 Results

### 3.1 Assessment of disease progression in an experimental cerebral malaria model

**Figure 1:**
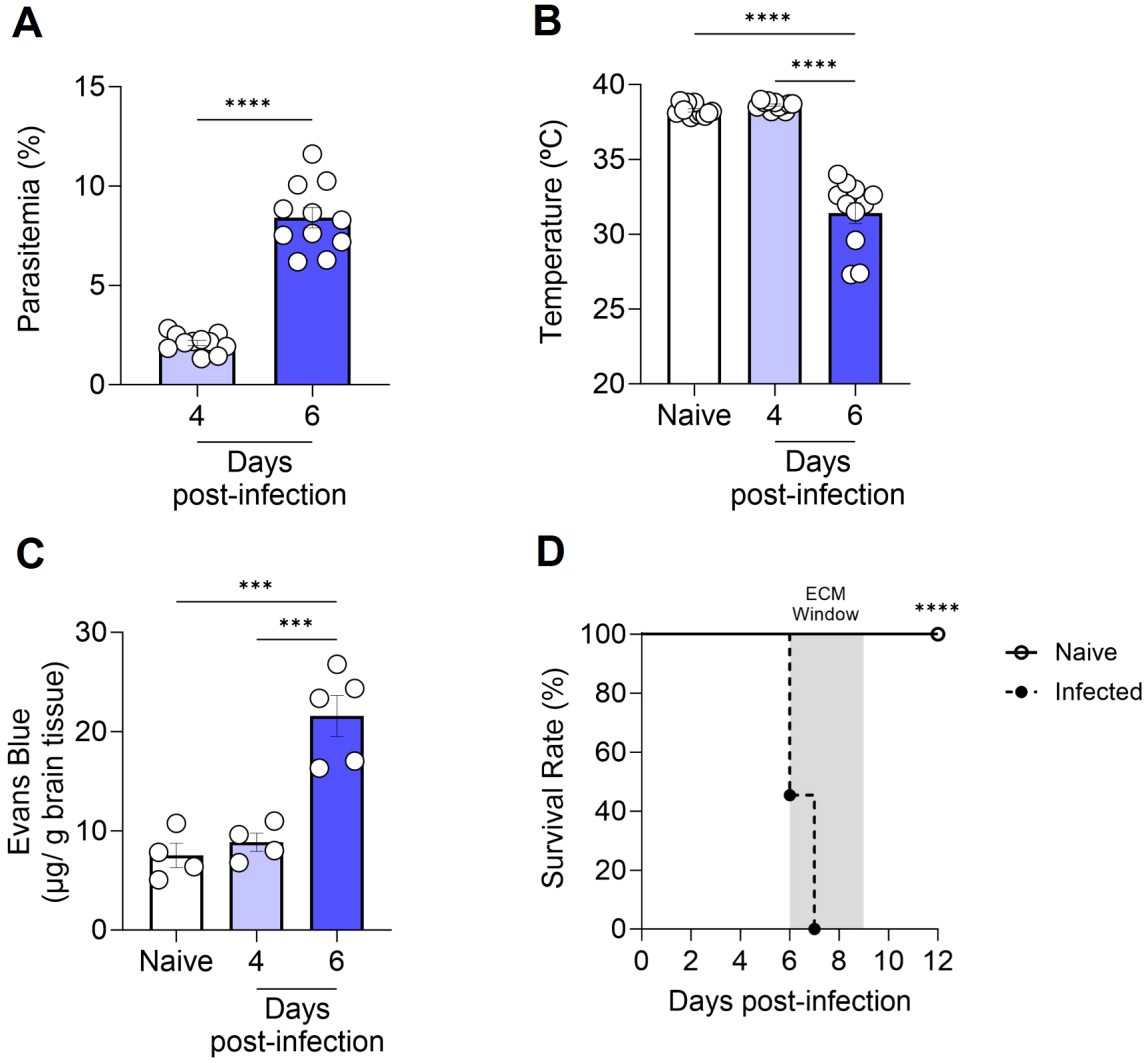
Progression of cerebral malaria in C57BL/6 mice infected with *Plasmodium berghei* ANKA. C57BL/6 mice were inoculated with 1x10^6^ iRBCs. Parasitemia (percentage of GFP^+^ cells) **(A)**, body temperature **(B)**, extravasation of Evans Blue dye to the brain parenchyma **(C)**, and survival rate **(D)** were assessed in naive and infected C57BL/6 mice at various time points. Significant differences between groups were analyzed using the following statistical tests: unpaired t-test **(A)**, one-way ANOVA **(B, C)**, and the Long-Rank test **(D)**. The results were statistically significant at p < 0.0006 (***) and p < 0.0001 (****). Graphs A and B: 10 mice per group. Graph C: 4–5 mice per group. Graph D: 10–22 mice per group. The data were pooled from two independent experiments **(A, B, D)** or are representative of two independent experiments **(C)**.

To corroborate prior literature data on the susceptibility of C57BL/6 mice to the development of ECM, we inoculated the animals with 1 × 10^6^ P*. berghei* ANKA-iRBCs and evaluated them at days 4 and 6 post-infection. The parasitemia of all infected C57BL/6 mice increased progressively (**Figure 1A**), accompanied by significant hypothermia (**Figure 1B**) and a breakdown of the BBB (**Figure 1C**) at day 6 post-infection, consistent with the development of ECM. In our experimental setting, infection of C57BL/6 mice with *P. berghei* ANKA resulted in 100% mortality between days 6 and 9 post-infection (**Figure 1D**).

### 3.2 Dynamics of cell populations in the brain during experimental cerebral malaria

**Figure 2:**
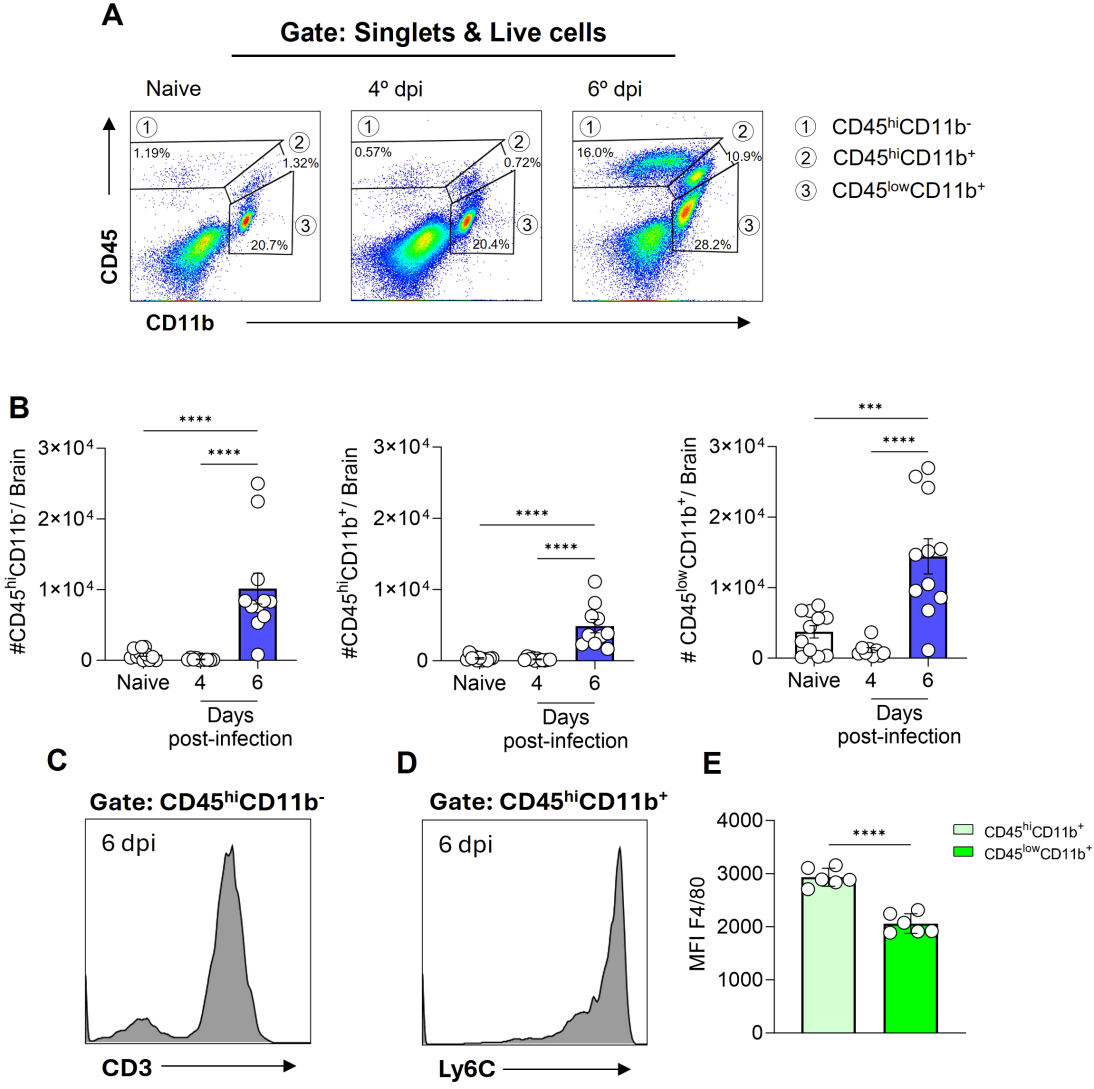
*Plasmodium berghei* ANKA infection alters the dynamics of cell populations in the mouse brain during ECM. C57BL/6 mice were inoculated with 1x10^6^ iRBCs. Representative dot plot of CD45 and CD11b cells, gated on live cells, in naive animal and at different stages of infection **(A)**. Absolute numbers of CD45^hi^CD11b^-^, CD45^hi^CD11b^+^ and CD45^low^CD11b^+^ cells **(B)**. Representative histograms illustrating the expression of CD3 on CD45^hi^CD11b^-^ cells **(C)** and Ly6C on CD45^hi^CD11b^+^ cells **(D)** on day 6 post-infection. Median fluorescence intensity (MFI) of F4/80 on the CD45^hi^CD11b^+^ and CD45^low^CD11b^+^ cells **(E)**. Significant differences between the groups were analyzed by one-way ANOVA with results indicated for p = 0.0002 (***) and p < 0.0001 (****). The number of mice per group ranged from 9 to 11. The data were obtained from a pool of two independent experiments.

To identify the leukocyte populations present in the cerebral environment during homeostasis and those that infiltrate during ECM development, flow cytometry analyses were conducted. The expression profile of the markers CD45 (expressed in various leukocytes) and CD11b (expressed in myeloid-lineage cells) enable the identification of three distinct cell populations within the brain tissue. These cell populations were designated as CD45^hi^CD11b^-^ cells (region 1), CD45^hi^CD11b^+^ cells (region 2), and CD45^low^CD11b^+^ cells (region 3) (**Figure 2A**). In naive animals, a pronounced presence of CD45^low^CD11b^+^ cells was observed, representing 15-25% of the cells. Additionally, CD45^hi^CD11b^+^ cells (0.4-2%) and CD45^hi^CD11b^-^ cells (0.5-2.5%) were present to a lesser extent (**Figure 2A**). Following infection with *P. berghei* ANKA, the numbers of these cell populations remained constant at day 4 post-infection (**Figure 2B**), a time point preceding the development of clinical signs of ECM (**Figure 1B**). However, at day 6 post-infection, a change in the dynamics of these cells occurred, characterized by a significant increase in the number of these three cell populations (**Figure 2B**).

The notable expansion of the CD45^hi^CD11b^-^ cell population, representing 15 to 25% of brain cells by day 6 post-infection, prompted further investigation into additional markers for enhanced identification. To specifically characterize this subset, we included CD3, a definitive T cell marker (**Figure 2C**, **Supplementary Figure S2B**), and Ly6C, also known as lymphocyte-6 antigen (**Supplementary Figure S1**) in our analysis. The Ly6C molecule is frequently expressed by specific subpopulations of T cells, monocytes and neutrophils (32, 33). A high proportion of the CD45^hi^CD11b^-^ cells expressed both CD3 and Ly6C, indicating that this particular cell subset, which increases in the brain at day 6 post-infection is predominantly formed by Ly6C^+^ T cells.

On the other hand, a distinct Ly6C expression (**Figure 2D**) was evident in the CD45^hi^CD11b^+^ cell population, while CD3 was not expressed (**Supplementary Figure S1**). Furthermore, our findings revealed that both CD45^hi^CD11b^+^ and CD45^low^CD11b^+^ cells are F4/80^+^ (a marker of cells of monocytic origin), as the majority of cells within these populations express this molecule (**Supplementary Figure S1D**). However, a notable distinction in expression levels was observed. In particular, the CD45^hi^CD11b^+^ cell population displayed a markedly elevated level of F4/80 molecule expression on their surface, in comparison to the CD45^low^CD11b^+^ cell population (**Figure 2E**).

### 3.3 Phenotypic characterization of brain myeloid cell populations in homeostasis and experimental cerebral malaria

To provide a more comprehensive characterization of the myeloid cell populations (CD45^hi^CD11b^+^ and CD45^low^CD11b^+^ cells), we conducted an analysis utilizing the cellular markers TMEM119 and CD206. Previous studies have demonstrated that under homeostatic conditions, microglia exhibit elevated levels of TMEM119 expression (34) and low expression of CD206, a mannose receptor (35).

**Figure 3:**
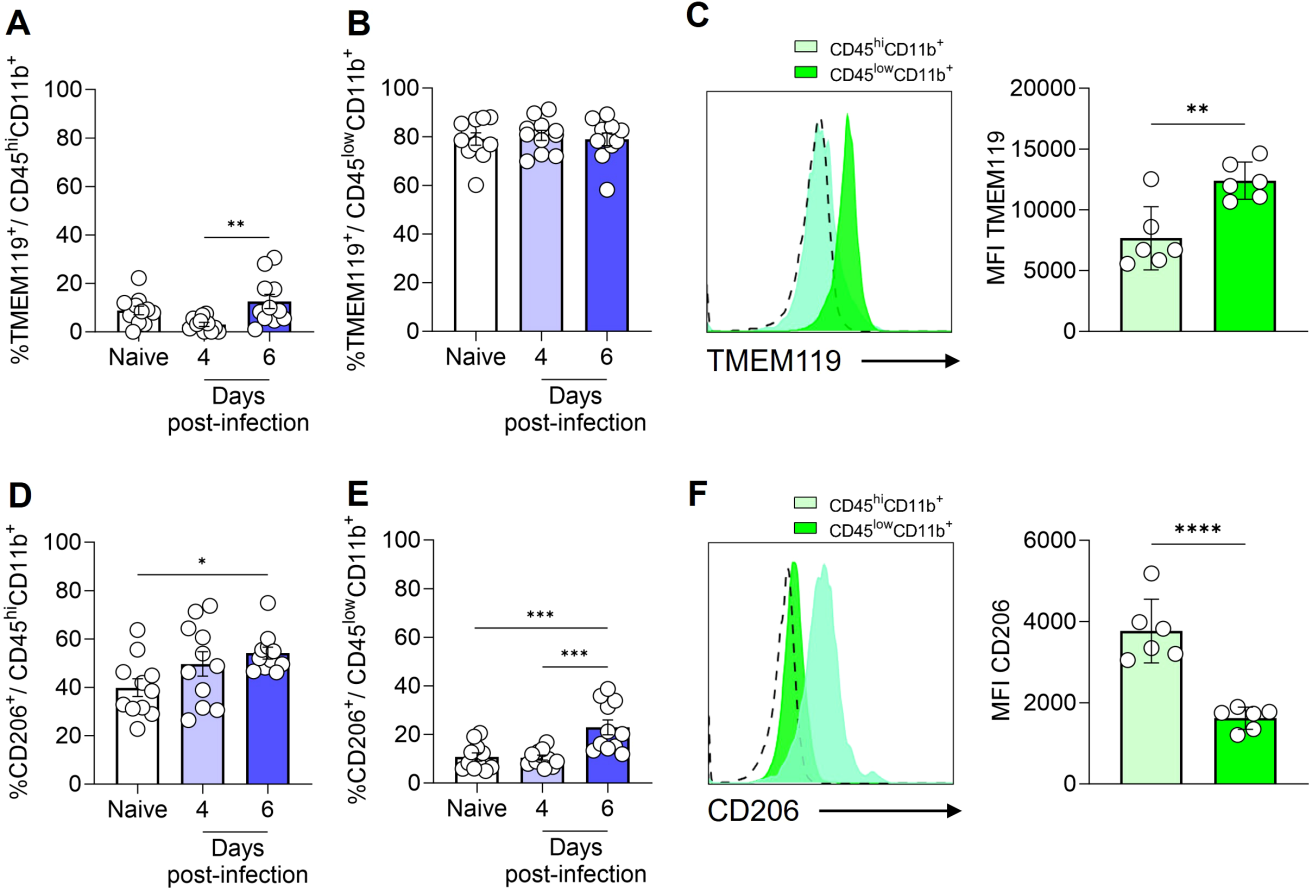
Expression of TMEM119 and CD206 by monocytes/macrophages and microglia. C57BL/6 mice were inoculated with 1x10^6^ iRBCs. Percentage of TMEM119^+^ cells within CD45^hi^CD11b^+^F4/80^+^ **(A)** and CD45^low^CD11b^+^F4/80^+^ **(B)** populations. Representative histogram and bar plots showing the median fluorescence intensity (MFI) of TMEM119 on the CD45^hi^CD11b^+^ and CD45^low^CD11b^+^ cells **(C)**. Percentage of CD206^+^ cells within CD45^hi^CD11b^+^F4/80^+^ **(D)** and CD45^low^CD11b^+^F4/80^+^ **(E)** populations. Representative histogram and bar plots showing the MFI of CD206 on the CD45^hi^CD11b^+^ and CD45^low^CD11b^+^ cells **(F)**. The dashed line in the histograms represents the FMO (fluorescence minus one) control. Significant differences between groups were analyzed by one-way ANOVA, with the results indicated for p = 0.03 (*), p = 0.007 (**), p = 0.0006 (***) and p < 0.0001 (****). The number of mice per group ranged from 9 to 11. The data were obtained from two independent experiments.

It was observed that a small fraction (less than 10%) of CD45^hi^CD11b^+^ cells exhibited the TMEM119 marker (**Figure 3A**). In contrast, CD206 expression was detected on nearly half of the CD45^hi^CD11b^+^ cells in naive animals (**Figure 3D**). By day 6 post-infection, a slight increase was observed in the percentage of CD45^hi^CD11b^+^ cells expressing CD206. In the meantime, the majority of CD45^low^CD11b^+^ cells expressed the TMEM119 marker (**Figure 3B**), with these values remaining consistent throughout the infection. Approximately 10% of CD45^low^CD11b^+^ cells in naive mice expressed the CD206 marker, with a slight increase observed by day 6 post-infection (**Figure 3E**). The mean fluorescence intensity (MFI), as illustrated by representative histograms and by bar plots overlaid with individual data points for TMEM119 and CD206, highlights statistically significant differences in expression between the cell populations. In CD45^low^CD11b^+^ cells, TMEM119 expression is predominant, while in CD45^hi^CD11b^+^ cells, CD206 expression is more significant (**Figures 3C, F**).

**Figure 4:**
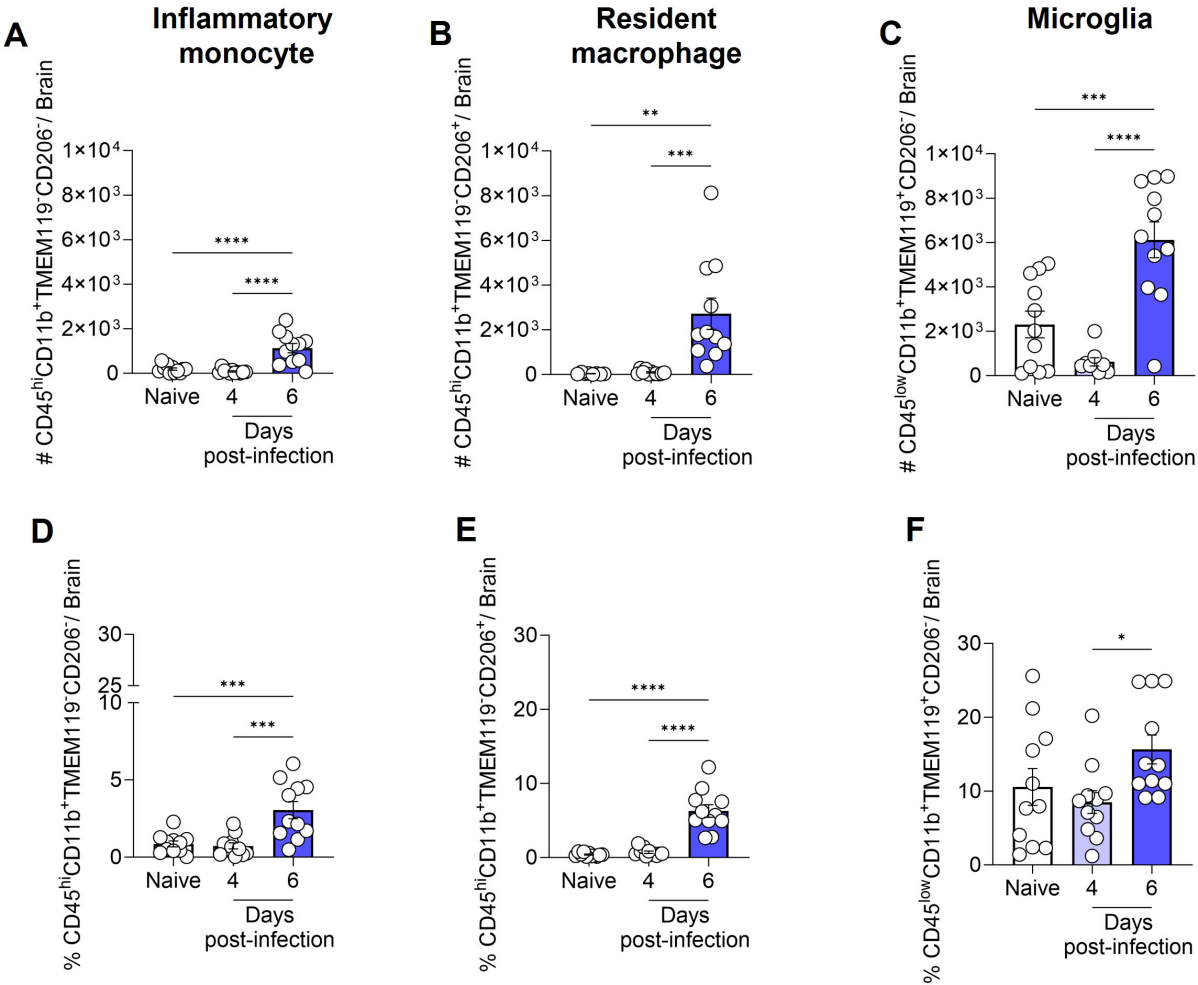
Total number and percentage of inflammatory monocytes, resident macrophages and microglia in the mouse brain following infection with *Plasmodium berghei* ANKA. C57BL/6 mice were inoculated with 1x10^6^ iRBCs. The absolute number **(A-C)** and percentage **(D-F)** of inflammatory monocytes (CD45^hi^CD11b^+^TMEM119^-^CD206^-^), resident macrophages (CD45^hi^CD11b^+^TMEM119^-^CD206^+^), and microglia (CD45^low^CD11b^+^TMEM119^+^CD206^-^). Significant differences between groups were analyzed by one-way ANOVA, with results indicated for p < 0.04 (*), p < 0.001 (**), p < 0.0005 (***), and p < 0.0001 (****). The number of mice per group ranged from 8 to 11. The data were obtained from a pool of two independent experiments.

Therefore, based on our analyses and the findings of previous literature (36, 37), we identified (**Supplementary Figure S2**) and quantified the total number (**Figures 4A–C**) and percentage (**Figures 4D–F**) of inflammatory monocytes (CD45^hi^CD11b^+^TMEM119^-^CD206^-^), resident macrophages (CD45^hi^CD11b^+^TMEM119^-^CD206^+^) and microglia (CD45^low^CD11b^+^TMEM119^+^CD206^-^) in the brain of naive and infected animals. This refined characterization, based on the expression or not of CD206 and TMEM, indicates that by day 6 of ECM development in *P. berghei* ANKA-infected mice, the number of microglia rose from 2 × 10^3^ to 6 × 10^3^ cells, representing a dramatic 300% expansion (**Figure 4C**). Additionally, the number of inflammatory monocytes and resident macrophages in the brain exhibited a significant increase, from a minimal quantity to 1 × 10^3^ cells (inflammatory monocytes; **Figure 4A**) and 2.7 × 10^3^ cells (resident macrophages; **Figure 4B**). The percentage of inflammatory monocytes (**Figure 4D**) and resident macrophages (**Figure 4E**) followed a similar trend, corresponding to the observed changes in cell numbers. However, the percentage elevation of microglia (**Figure 4F**) was less pronounced, likely attributable to the concurrent increase in other cell types within the brain. These findings indicate that, at the onset of ECM, the brain experiences a robust inflammatory response.

### 3.4 IL-1β, TNF and iNOS expression by inflammatory monocytes, resident macrophages and microglia

**Figure 5:**
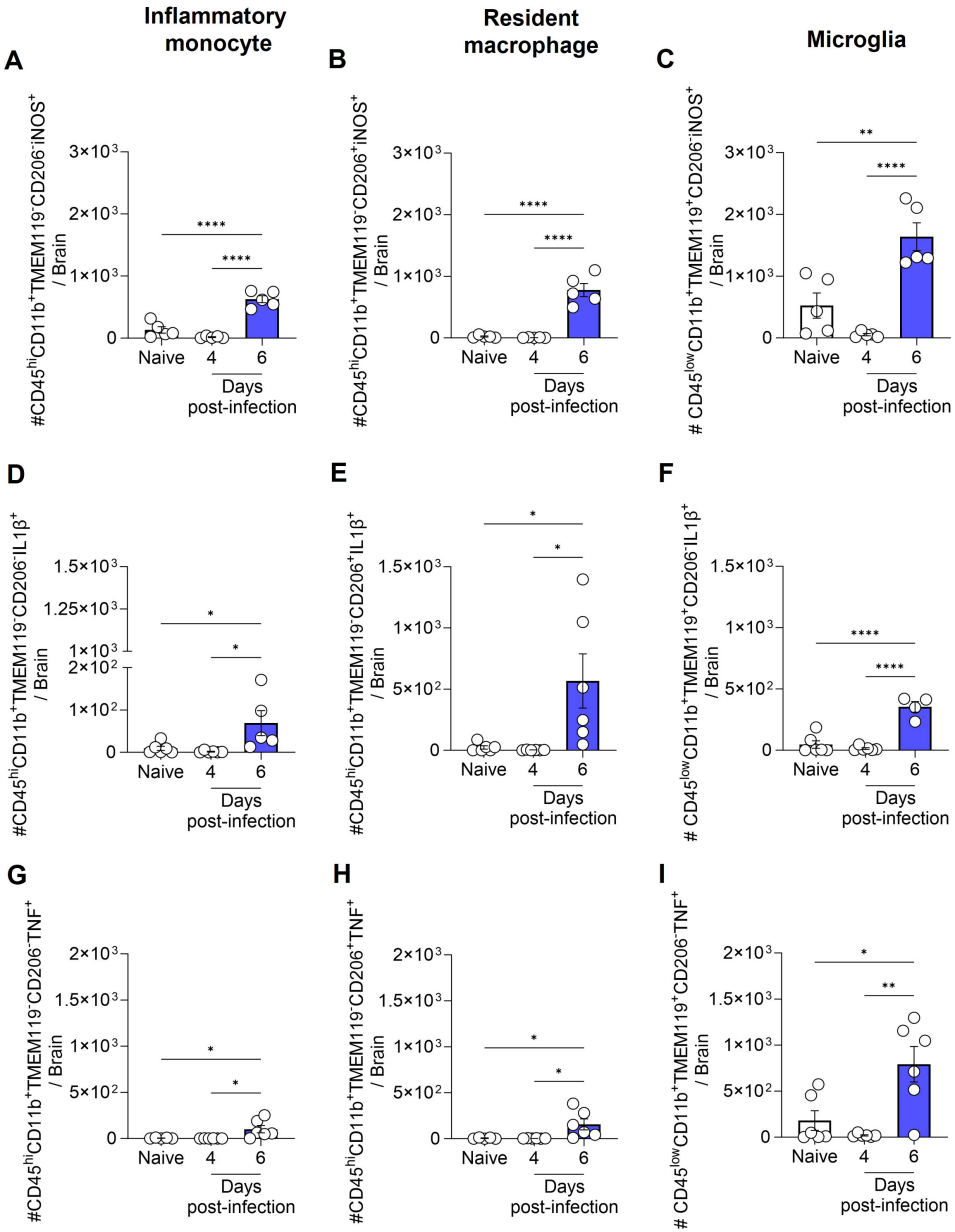
Total number of inflammatory monocytes, resident macrophages and microglia expressing iNOS, and producing IL-1β or TNF during ECM. C57BL/6 mice were inoculated with 1x10^6^ iRBCs. The absolute number of iNOS^+^ **(A-C)**, IL-1β^+^ **(D-F)**, and TNF^+^ **(G-I)** cells among inflammatory monocytes, resident macrophages and microglia. Significant differences between groups were analyzed by one-way ANOVA, with results indicated for p < 0.03 (*), p < 0.002 (**), and p < 0.0001 (****). The number of mice per group ranged from 4 to 6. Representative data from two independent experiments.

Next, we assessed the production of IL-1β, TNF, and the expression of iNOS by inflammatory monocytes (CD45^hi^CD11b^+^TMEM119^-^CD206^-^), resident macrophages (CD45^hi^CD11b^+^TMEM119^-^CD206^+^), and microglia (CD45^low^CD11b^+^TMEM119^+^CD206^-^) (**Supplementary Figure S3**). On day 6 post-infection, the increase in the total number of inflammatory monocytes, resident macrophages, and microglia, as previously described in **Figures 4A–C**, was accompanied by a rise in the number of cells from these same populations expressing iNOS (**Figures 5A–C**), and producing IL-1β (**Figures 5D–F**) or TNF (**Figures 5G–I**).

It is important to note that while the total number of iNOS^+^, IL-1β^+^, and TNF^+^ inflammatory monocytes, resident macrophages and microglia increased over the course of infection, the percentage of iNOS^+^ cells among each population (inflammatory monocytes, macrophages and microglia) either remained constant or decreased (**Supplementary Figure S4A-C**). Conversely, the percentage of IL-1β^+^ and TNF^+^ microglia increased by day 6 post-infection (**Supplementary Figure S4F, I**). However, given the low number of inflammatory monocytes and resident macrophages in the brains of naive animals, which resulted in high variability in the percentage analysis, no significant difference was observed in the percentage of IL-1β^+^ and TNF^+^ inflammatory monocytes and resident macrophages between naive and infected animals (**Supplementary Figure S4D, E, G, H**).

## 4 Discussion

The data presented herein provide knowledge of the dynamics of the inflammatory response of mononuclear cells in the CNS, including microglia and other populations associated with and recruited to the brain during *P. berghei* ANKA infection of C57BL/6 mice, an experimental model of ECM.

The initial investigations into microglia activation during CM in humans (38) and in experimental models (28) were conducted in the late 1990s and revealed several important aspects, including local inflammation, which should be considered in the quest to understand the pathophysiological mechanisms of CM. In these studies, microglia activation was described through changes in their morphology and expression of MRP8 and MRP14, calcium-binding sensor proteins of activated monocytes. At that time, the identification of the cell of interest was achieved through the use of classical histology and immunohistochemistry, employing markers that are currently known to be inadequate for distinguishing microglia from other resident and recruited CNS mononuclear cells (28, 38).

Two additional studies from the mid-2000s employing distinct methodologies have corroborated the activation of microglia during ECM. The initial study employed automated histological analysis, which considered Iba1 expression and morphological changes in its conclusions (39). The second study utilized transcriptomic analysis of microglia isolated from the brains of C57BL/6 mice infected with *P. berghei* ANKA. This analysis was conducted at two distinct time points, prior to and during the clinical manifestations of ECM. The results demonstrated that genes are differentially expressed at these two time points following infection (29).

In the present study, a combination of distinct cell markers was employed to identify specific cell populations in the brains of C57BL/6 mice infected with *P. berghei* ANKA, utilizing flow cytometry. The differential expression of the CD45 and CD11b molecules enable the identification of distinct cell populations. The observed differences in CD45 expression levels (CD45^hi^ and CD45^low^) between myeloid cells (CD11b^+^) indicate the existence of two distinct myeloid cell populations. However, previous studies have indicated that CD45 expression can be altered during infections (40, 41), and thus we included additional markers to more accurately identify and characterize these cells. These included the transmembrane protein TMEM119 and the mannose receptor CD206.

Bennett et al. (42) demonstrated that TMEM119 is exclusively expressed by microglia during homeostasis in both humans and mice. This protein has been identified as a potential microglial marker that is capable of differentiating resident microglia from blood-derived macrophages in the brain (42). This finding has been corroborated by several subsequent studies (43–46). Furthermore, Hattori et al. (37) employed the molecule CD206 to distinguish microglia (CD206^-^ cells) from resident macrophages (CD206^+^ cells) (37), a strategy also employed in other studies (36, 47, 48).

The combination of the aforementioned markers with more traditional ones revealed that the majority of CD45^low^CD11b^+^ cells were TMEM119^+^, and this population was identified as microglia. Conversely, CD45^hi^CD11b^+^ cells were predominantly negative for TMEM119. A subset of these TMEM119^-^ cells were also CD206^-^, thus classifying them as inflammatory monocytes, while the remaining subset expressed CD206, resulting in their classification as resident macrophages. Following the precise characterization of these three mononuclear phagocyte populations, the findings indicate that C57BL/6 animals infected with *P. berghei* ANKA exhibit an influx of inflammatory monocytes from the periphery to the brain, detectable on day 6 post-infection. Furthermore, a notable increase in the overall number of resident macrophages and microglia was observed at this same time, indicating that all these cell types can sense and respond dynamically to the infectious process, potentially contributing to the local immune landscape during ECM progression. However, while the present study provides unequivocal evidence of the expansion of these CNS-resident and infiltrating phagocytes during ECM, it does not establish a causal link between these changes and disease pathogenesis. This is acknowledged as a limitation of the current study. Upcoming experimental approaches employing depletion or inhibition strategies (e.g., clodronate liposomes or genetic models) at the correct stage of disease will be essential to determine the functional contribution of these cell populations to ECM progression.

The observed increase in the number of microglia in the acute phase of ECM may be attributed to the proliferation of these cells. These findings are consistent with those previously reported by Capuccini et al. (29), who observed an increase in the expression of genes related to cell proliferation before the onset of ECM, as well as an increase in the number of these cells in the brain during ECM. At the onset of ECM clinical signals, the transcriptomic analysis also demonstrated an upregulation of genes involved in the immune response and chemokine production (29).

Data from our experimental model revealed that, in addition to inflammatory monocytes and resident macrophages, microglia also exhibited a significant increase in the total number of IL-1β^+^ and TNF^+^ cells at day 6 post-infection, thereby highlighting the activation of pro-inflammatory pathways within the CNS. The production of IL-1β and TNF in the brain during *Plasmodium* infection has important functional implications for the pathogenesis of both ECM and CM. In murine models, TNF is essential for endothelial activation, BBB breakdown, and neuroinflammation, largely through upregulation of ICAM-1/VCAM-1 and recruitment of leukocytes to brain microvessels (49–51). Although IL-1β is not required for ECM development (52), it may exacerbate vascular permeability and inflammatory signaling once inflammation is established (53). In human CM, elevated levels of TNF and IL-1β have been found in brain tissue and plasma, correlating with vascular damage, immune cell infiltration, and neuronal injury (54, 55). These cytokines likely contribute to both endothelial dysfunction and neuronal pathology in CM, reinforcing their relevance as potential targets for therapeutic intervention.

Interestingly, studies conducted in mice deficient in NLRP3, caspase-1, the adaptor protein ASC, or the IL-1 receptor demonstrated that these mice exhibited comparable disease outcomes to wild-type mice, indicating that the activation of the inflammasome pathway does not play a significant role in the immunopathology caused by *P. berghei* ANKA (52, 56). However, Strangward P. et al. (57) observed that IL-33 therapy, in conjunction with antimalarial drugs, selectively inhibited the NLRP3-IL-1β inflammasome axis in microglia and monocytes, resulting in a significant reduction in IL-1β production in both cell types (57). This, in turn, led to an improvement in the treatment success of established ECM (57). In addition, a recent study showed that mice with combined deficiencies of caspases-8/1/11 or caspase-8/gasdermin-D (GSDM-D) exhibited impaired capacity to produce both TNF and IL-1β, and demonstrated high resistance to the development of ECM (58).

Despite the observed increase in the absolute number and percentage of IL-1β- and TNF-producing microglia throughout the course of infection, as well as an increase in the absolute number of cells expressing iNOS, the inducible form of the nitric oxide (NO)-producing enzyme, the percentage of cells expressing iNOS was found to be reduced. NO plays a fundamental role in the functioning of the brain, regulating blood flow and maintaining vascular integrity (59, 60). Furthermore, NO prevents the excessive adhesion of leukocytes and platelet aggregation in cerebral microvasculature, thereby preventing blockages and inflammation (61–63).

Nevertheless, the role of NO in malaria remains a topic of debate (64). A number of studies have demonstrated that the production of NO by immune cells is of significant importance in regulating the blood-stage parasite (65, 66), although it may potentially contribute to the development of CM (67). In contrast, it appears that in African children with malaria, NO exerts a protective effect rather than contributing to pathology (68). This observation is further supported by *in vivo* experiments conducted on mice deficient in iNOS or eNOS, which indicated that the low bioavailability of NO is associated with the development of ECM. The iNOS^-/-^ or eNOS^-/-^ animals exhibited parasitemia and ECM development course comparable to that of the control group (69, 70), while administration of exogenous NO protected the animals from ECM (63, 70). Indeed, several mechanisms have been described in malaria that result in a decrease in the bioavailability of NO (70, 71).

Curiously, our observation of the relative reduction (percentage) of iNOS expression in microglia throughout infection may be directly correlated with increased proliferation during ECM development. This hypothesis is consistent with the findings of Maksoud et al. (72), who describe that microglia exhibit basal iNOS activity and that the iNOS/NO signaling pathway inhibits microglial cell proliferation by activating protein kinase G (PKG) (72). However, this remains speculative and should be interpreted with caution. Validation of this potential mechanism will require experiments using proliferation markers, such as Ki-67.

Although not the primary focus of this study, we were able to observe a significant increase in the number of T cells (CD45^hi^CD11b^-^CD3^+^) expressing the lymphocyte-6 antigen (Ly6C) on their surface in the brain during the acute phase of ECM, a potentially novel and intriguing finding. The migration and adhesion of CD8 T cells to the cerebral vascular endothelium and their association with pathology have been previously described in CM and ECM (7, 15, 73). It is of interest to note that this is the first report, to the best of our knowledge, in which T cells expressing Ly6C have been observed in the brain during ECM. Our current analysis, however, did not determine the specific T cell lineage of these Ly6C^+^ T cells, but it is plausible that they belong to the CD8 T cell subset. Prior studies have established a correlation between Ly6C expression by CD8 T cells and augmented activation, cytotoxic effector function, and cytokine production (74–76). In particular, Kusaka et al. demonstrated that Ly6C^+^ CD8 T cells constitute a major source of IFN-γ during *Legionella pneumophila* infection, thereby emphasizing their role in regulating the inflammatory response. Prospective studies employing additional markers such as CD8, combined with functional assays to evaluate cytokine secretion and cytotoxic activity, will be essential to determine whether this subset of Ly6C^+^ T cells contributes to immunopathology in ECM. Such analyses may provide key insights into the cellular mechanisms underlying neuroinflammation and vascular damage in ECM, and open the opportunity to explore this population in the context of human CM.In conclusion, our findings revealed a notable elevation in the number of distinct immune cells, indicating their reactivity to *P. berghei* ANKA infection, which may be associated with the pathogenesis of ECM. Furthermore, we underscore the activation of microglia through an increase in the total number of cells expressing iNOS and producing TNF or IL-1β. Collectively, our results provide valuable insights into the potential role of microglial reactivity in contributing to neuroinflammation during ECM. Future studies should focus on analyzing intermediate time points, such as days 5 and 5.5 post-infection, and employ monocyte and microglia depletion strategies to delineate their specific roles in the pathogenesis of ECM.

## Data Availability

The raw data supporting the conclusions of this article will be made available by the authors, without undue reservation.
