# Supplementary material for: Microglia exhibit a dynamic response, modulating inducible nitric oxide synthase expression and the production of pro-inflammatory cytokines during experimental cerebral malaria
Source: Front Immunol. 2025 Jul 23;16:1494418. doi: 10.3389/fimmu.2025.1494418 (PMC12325256; doi:10.3389/fimmu.2025.1494418)
Supplement: Supplementary file 1 [file DataSheet1.docx]

Supplementary Material

# Supplementary Figure

**
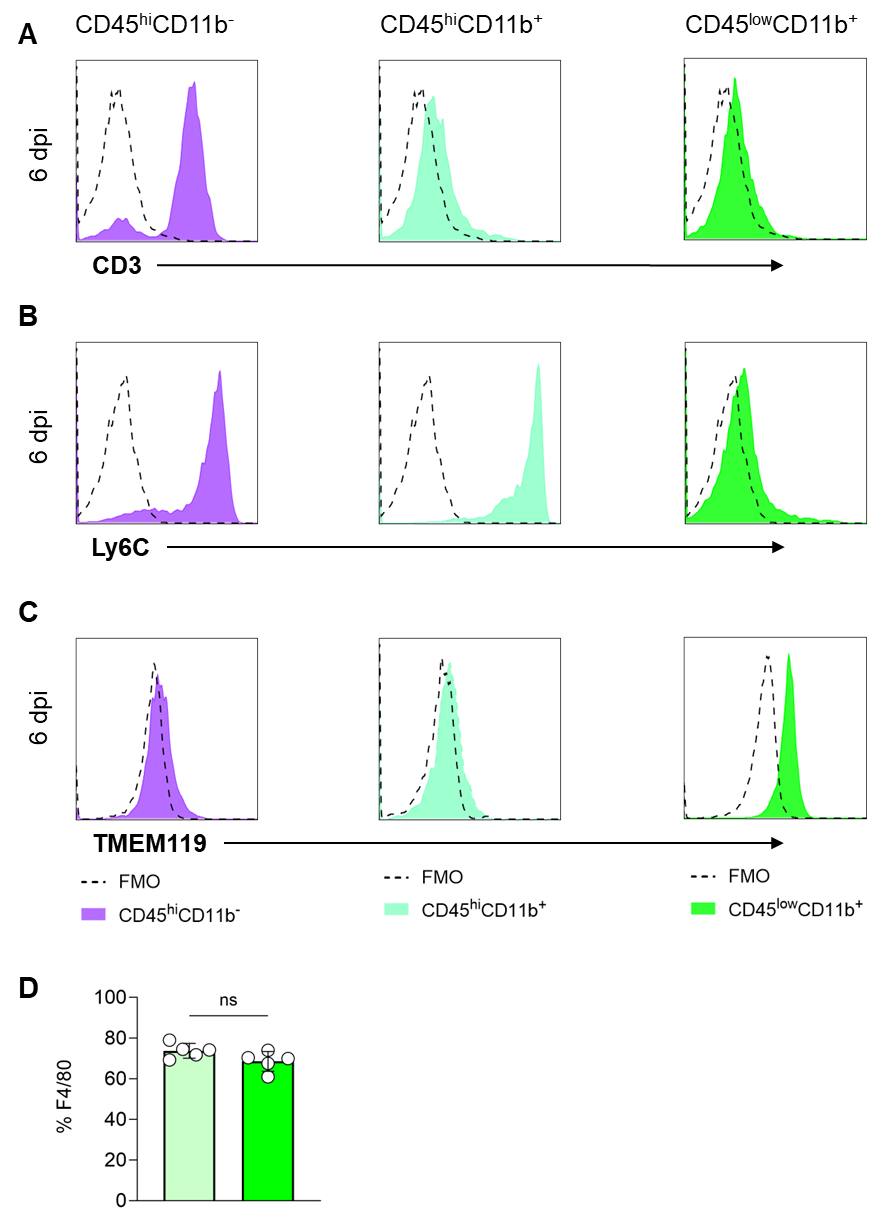
**

Supplementary Figure 1. Differentiation of T cells, monocytes/macrophages and microglia in the brain of C57BL/6 animals with ECM through the expression of specific markers. C57BL/6 mice were inoculated with 1x10^6^ iRBCs. Representative histograms of CD3 (A), Ly6C (B) and TMEM119 (C) expression on the surface of the CD45^hi^CD11b^-^, CD45^hi^CD11b^+^ and CD45^low^CD11b^+^ cell populations on day 6 post-infection. The negative control (FMO) is represented by a dotted line. (D) Percentage of cells F4/80^+^ among the CD45^hi^CD11b^+^ and CD45l^ow^CD11b^+^ cells.

# Supplementary Figure


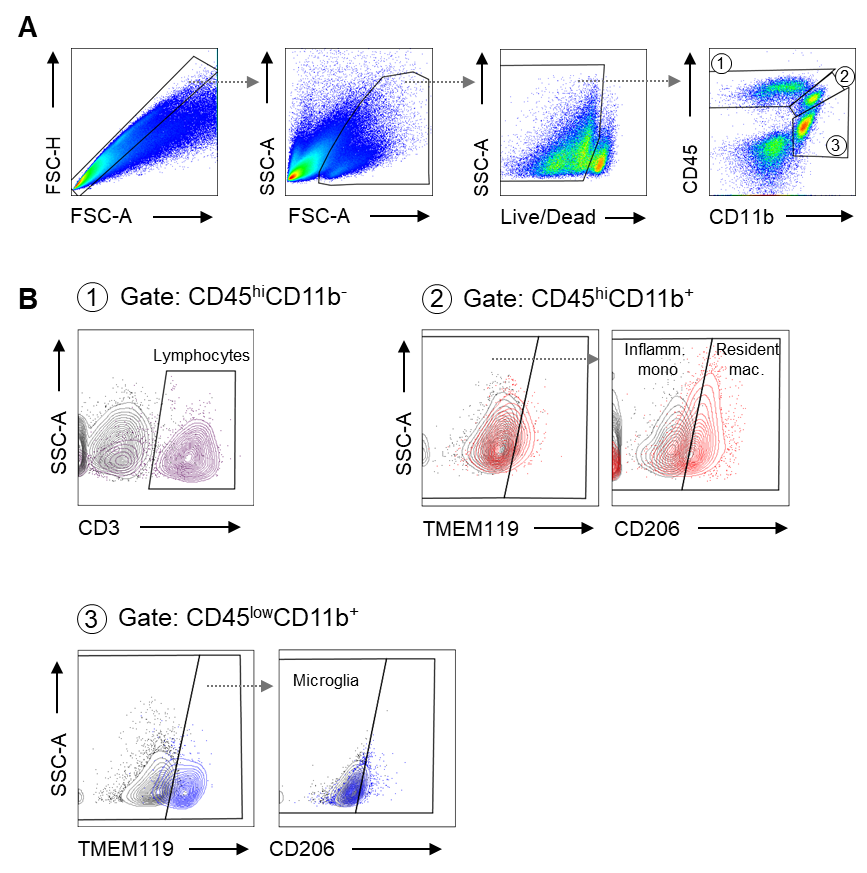


Supplementary figure 2. Representative gating strategy used to identify the three major subsets based on CD45 and CD11b expression, as well as lymphocytes, inflammatory monocytes, resident macrophages and microglia. Representative plots illustrating the gating strategy employed for the analysis of brain tissue cells. Following the gating on singlets and live cells, three cell subpopulations were notorious based on CD45 and CD11b expression (A). Among the CD45^hi^CD11b^-^, CD3^+^ cells were identified as lymphocytes. Among the CD45^hi^CD11b⁺ population, further characterization using TMEM119 and CD206 expression allowed the identification of inflammatory monocytes (CD45^hi^CD11b⁺TMEM119⁻CD206⁻) and resident macrophages (CD45^hi^CD11b⁺TMEM119⁻CD206⁺). Within the CD45^low^CD11b⁺ population, TMEM119⁺ cells were identified as microglia (CD45^low^CD11b⁺TMEM119⁺CD206⁻) (B). The identification of positive populations was facilitated by employing the fluorescence minus one (FMO) control, represented in the gray contour plot, as a negative reference.

# Supplementary Figure


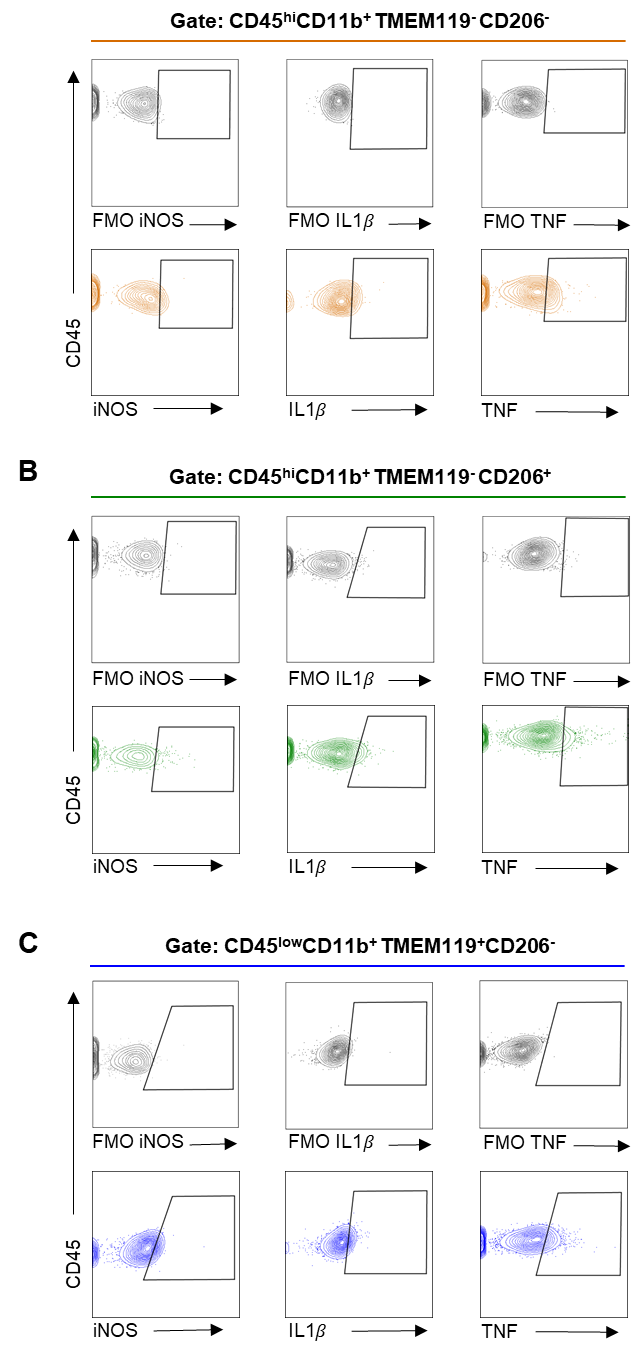


Supplementary figure 3. Gating strategy used to identify the expression of iNOS and the production of cytokines by inflammatory monocytes, resident macrophages and microglia. Representative flow plots to illustrates the analysis of of iNOS^+^, IL1β^+^ and TNF^+^ populations among inflammatory monocytes (CD45^hi^CD11b^+^TMEM119^-^CD206^-^) (A), resident macrophages (CD45^hi^CD11b^+^TMEM119^-^CD206^+^) (B) and, microglia (CD45^low^CD11b^+^TMEM119^+^CD206^-^) (C). The identification of positive populations was facilitated by employing the fluorescence minus one (FMO) control as a negative reference.

# Supplementary Figure


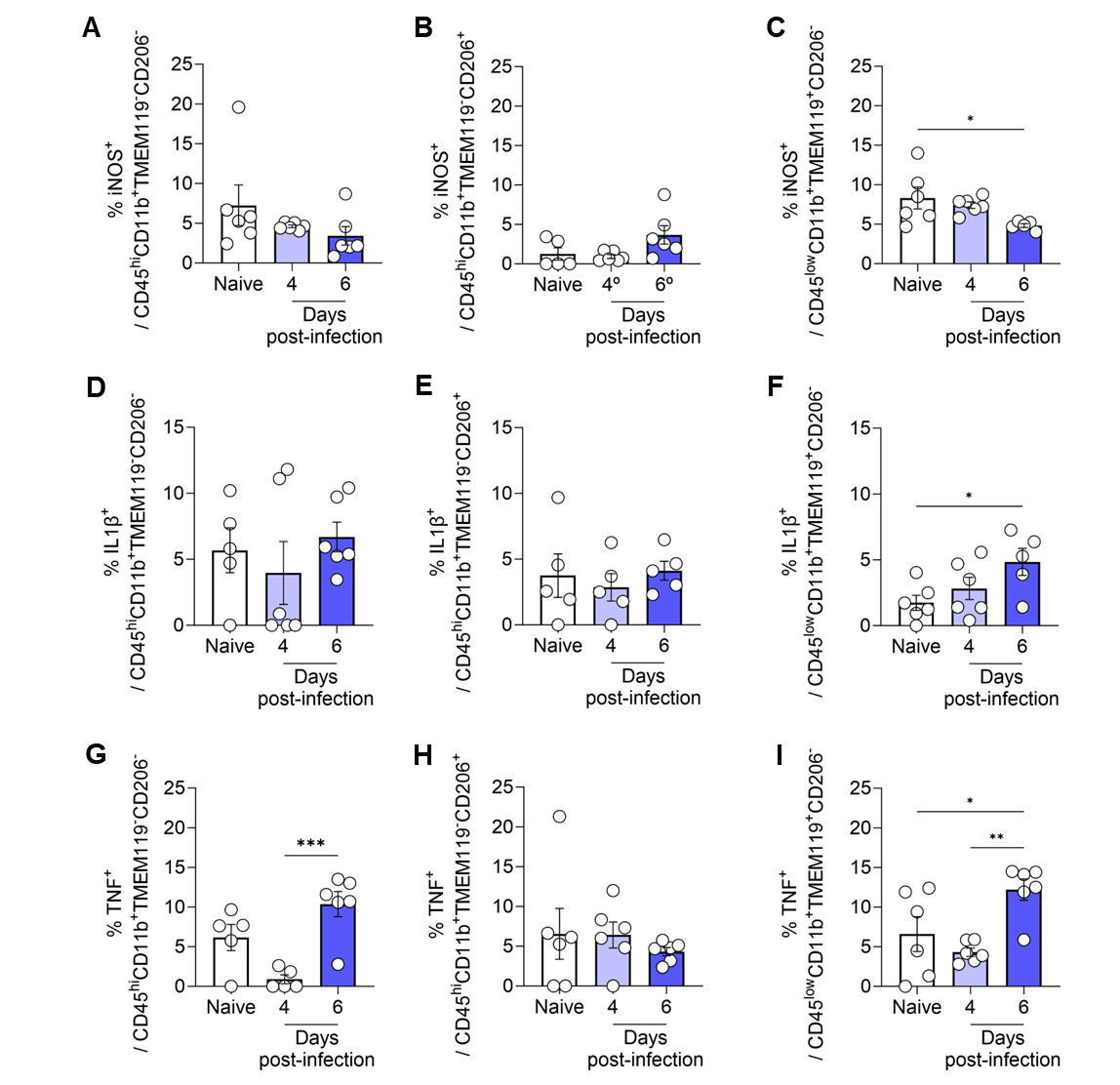


Supplementary figure 4. Relative frequency of iNOS, IL-1β and TNF expression by inflammatory monocytes, resident macrophages and microglia during ECM. C57BL/6 mice were inoculated with 1x10^6^ iRBCs. Percentage of iNOS^+^ (A-C), IL-1β^+^ (D-F) and TNF^+^ (G-I) cells within the inflammatory monocytes, resident macrophages and microglia. Significant differences between groups were analyzed by one-way ANOVA, with results indicated for p < 0.04 (*), p < 0.006 (**) and p < 0.0009 (***). The number of mice per group ranged from 5 to 6. Representative data from two independent experiments.
